# Immune and Imaging Correlates of Mild Cognitive Impairment Conversion to Alzheimer’s Disease

**DOI:** 10.1038/s41598-017-16754-y

**Published:** 2017-12-01

**Authors:** Francesca La Rosa, Marina Saresella, Francesca Baglio, Federica Piancone, Ivana Marventano, Elena Calabrese, Raffaello Nemni, Enrico Ripamonti, Monia Cabinio, Mario Clerici

**Affiliations:** 1 Laboratory of Molecular Medicine and Biotechnology, Don Gnocchi Foundation, IRCCS, Milan, Italy., Milan, Italy; 2 Department of Physiopathology and Transplants, University of Milano, 20100 Milan, Italy

## Abstract

Amnestic mild cognitive impairment (aMCI) conversion to Alzheimer’s disease (AD) is seen in a sizable portion of aMCI patients; correlates predicting such conversion are poorly defined but neuroinflammation and the reactivation of chronic viral infections are suspected to play a role in this phenomenon. We analyzed these aspects in two homogeneous groups of aMCI who did or did not convert to AD over a 24-months period. Results showed that at baseline in those aMCI individuals who did not convert to AD: 1) Aβ_1-42_ stimulated production of the pro-inflammatory cytokines IL6 and IL1β by CD14^+^ cells was significantly reduced (p = 0.01), 2) CD14^+^/IL-33^+^ cells were increased (p = 0.0004); 3) MFI of TLR8 and TLR9 was significantly increased, and 4) better preserved hippocampus volumes were observed and correlated with IL33^+^/CD14^+^ cells. Notably, Aβ_1-42_ stimulated production of the antiviral cytokine IFN-λ was increased as well in non-AD converters, although with a borderline statistical significance (p = 0.05). Data herein indicating that proinflammatory cytokines are reduced, whereas IFN-λ production and TLR8 and 9 MFI are augmented in those aMCI in whom AD conversion is not observed suggest that the ability to mount stronger antiviral response within an antiiflammatory milieu associates with lack of AD conversion.

## Introduction

Mild cognitive impairment (MCI) is defined as a subjective and objective decline in cognitive performance that is greater than expected for an individual’s age and education level, but does not meet criteria for the diagnosis of dementia^1–3^. Elderly MCI patients are nevertheless at high-risk for developing dementia and, in particular, Alzheimer’s disease (AD)^3–5^. Recently, the International Working Group (IWG) introduced the terminology of MCI to refer to individuals with cognitive impairment that is not as severe as compared to what is seen in AD patients^6^. This situation thus represents a borderline condition between normal aging and AD^7^. Notably, although the diagnostic accuracy has certainly increased, correlates of MCI conversion to AD are still poorly defined.

The estimated annual rate of MCI conversion to AD ranges between 10% and 15%^8^, and MCI individuals in whom memory loss is the predominant symptom (amnestic MCI -aMCI-) are more prone to progress to AD. Predictive studies have described a number of biological and cognitive factors, including cognitive reserve^9^, performance in cognitive testing^10^, and the presence and concentration of biomarkers in the cerebrospinal fluid (CSF)^11–13^; that are suggested to correlate with AD conversion. Volumetric magnetic resonance imaging (MRI)^14–16^ and fluorodeoxyglucose and Pittsburgh compound B positron emission tomography (FGD-PET, PIB-PET)^17,18^ can also be useful in order to recognize those patients in whom AD conversion is more likely to occur. In particular, hippocampal volume as measured with MRI advanced techniques is a well-known biomarker of downstream neural degeneration or injury^2^. Although not suitable for defining preclinical AD-stage if considered as unique index, this downstream topological biomarker is adequate for the screening of subjects at risk, as recently stressed^19^.

The validity of these results is nevertheless hampered by the fact that most of published studies utilized a single marker to predict progression to AD^9,10,14–16^ even if this phenomenon is multifactorial. Such multidimensionality,is reflected in the observation that a combination of MRI (hippocampal volumes)^14–16^, genetic (ApoE)^20^ and humoral (CSF levels of beta amyloid and phosporylated τ protein)^11–13^ indexes is currently suggested to have the greatest predictive value toward AD conversion.

Genetic and environmental factors interact in the pathogenesis of AD, a complex and still scarcely understood process in which viral infections, and in particular Human Herpes Simplex virus type 1 infection (HSV-1) are suggested to play a role^21–25^. Microbial infections stimulate innate immunity via binding of the pathogen associated molecular patterns (PAMPS) they express to toll like receptors (TLR). Nucleic acids produced upon viral replication, in particular, ligate TLR8 and 9^26,27^ result in the activation of innate immunity and the production of multiple cytokines, amongst which interferon-lambda (IFN-λ) plays a pivotal role in limiting viral replication and the infection of target cells^28^. Recent results indicating that TLR expression is increased in immune cells of MCI individuals^29,30^ led to hypothesize that stronger immune responses to viruses could be detected in these individuals.

We analyzed whether immune correlates that predict MCI conversion into AD could be identified analyzing immunological parameters in a cohort of aMCI patients in whom AD conversion was or was not observed over a 24-months period. In addition, we also analyzed one of the MRI biomarker of AD-associated neural injury, the hippocampal volume.

Results herein suggest that stronger antiviral responses in the absence of inflammation is correlated to better preservation of the hippocampus volume and could predict AD conversion.

## Results

### Clinical characteristics of the individuals enrolled in the study

Demographic and clinical characteristic of the individuals enrolled in the study are summarized in Table 1. As per inclusion criteria, no differences were observed in gender, age, years of education, global cognitive levels (MMSE) and ApoE4 status when AD converters and AD non-converters were compared at baseline.

**Table 1:** Demographic and clinical characteristics at baseline of the individuals enrolled in the study who had a diagnosis of amnestic mild cognitive impairment and did (AD converters) or did not (AD non-converters) convert to AD over a 24-months period. Mini-Mental State Evaluation (MMSE) after follow up indicates the value 24 months after the baseline result.

	AD converters	AD non-converters
Number (N)	25	21
Gender (M:F)	14:11	12:9
Age, years at baseline	76 ± 5.6	72 ± 6.2
Level of education, years	8.25 ± 2.71	7.62 ± 3.62
MMSE	25 ± 1.9	26 ± 2.1
MMSE after follow up	20 ± 5.1*^#^	25 ± 2.2
APOE ε-4 cariers (%)	20	20
L HV, mm^3^	2496 ± 560.2	3409.4 ± 402.5**^#^
R HV, mm^3^	2633.5 ± 492.6	3413.6 ± 496.4***^#^

*p = 0.003 between baseline and follow-up in AD-converters. **p = 0.04 at baseline between AD converters and AD non-converters. ***p = 0.01 at baseline between AD converters and AD non-converters. ^#^Power 1 − β ≥ 0.90 considering MMSE score or Hippocampal Volume (HV).

### TLR8 and TLR9 expressing CD14^+^ cells

**Figure 1:**
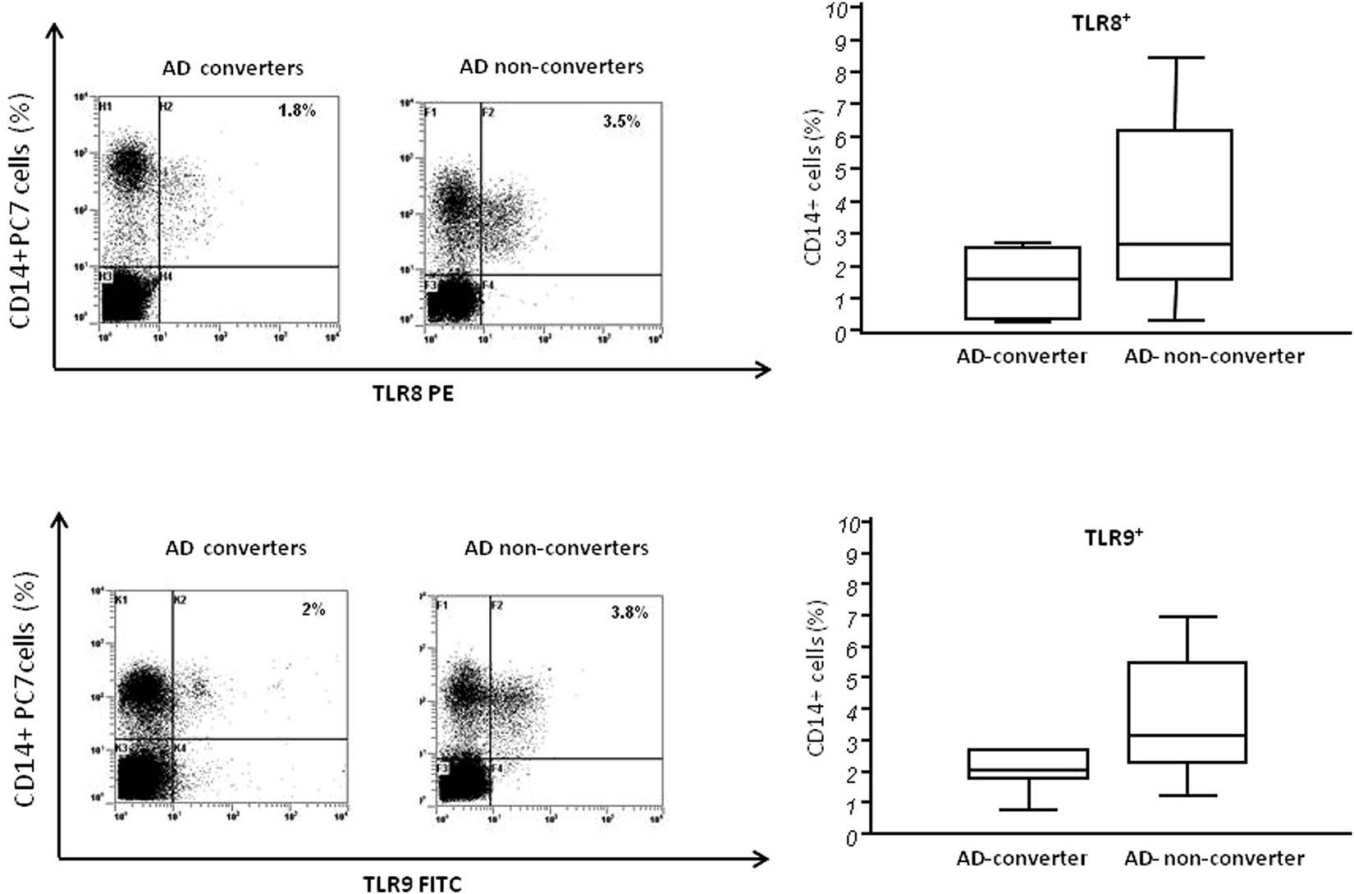
TLR8 and TLR9 expressing CD14+ cells: TLR8- (upper panels) and TLR9- (lower panels) expressing CD14^+^ cells upon stimulation with specific agonists (ODN and ssRNA, respectively). Representative results in MCI individuals who did (AD-converters) or did not (AD non-converters) convert to AD over a 24-months period are presented in the left part of the figure; in the upper right corner the percentage of CD14/TLR8^+^ and CD14/TLR9^+^ cells is shown. Summary results were obtained in 21 AD converters and 25 AD non-converters and are shown in the bar graphs. The boxes stretch from the 25 to the 75 percentile; the line across the boxes indicates the median values; the lines stretching from the boxes indicate extreme values. Outside values are displayed as separate points.

CD14+ immune cells of AD converters and AD non-converters were either unstimulated or stimulated with TLR8 and 9 agonists (ODN and ssRNA, respectively), and the percentage of TLR-expressing cells was examined by flow-cytometry. Results showed that, whilst no differences were seen in unstimulated conditions (<1% of CD14+ cells expressed TLR8 or TLR9; data not shown), agonist-stimulated TLR8- and TLR9-expressing CD14+ cells were augmented, albeit not significantly, in AD non-converters (medians: TLR8 = 2.9%, TLR9 = 3.1%) compared to AD converter (medians: TLR8 = 1.6%, TLR9 = 2%; p = 0.06 in both cases) (Fig. 1). Notably, though, TLR8 and TLR9 Mean Fluorescence Intensity (MFI) was significantly increased in AD non-converters compared to AD converters (p = 0.0004, and p = 0.0001, respectively) (Table 2).

**Table 2:** Agonist-stimulated TLR8 and TLR9 mean fluorescence intensity (MFI) at baseline in CD14^+^ cells of patients with a diagnosis of amnestic mild cognitive impairment who did (AD converters) or did not (AD non-converters) progress to Alzheimer’s disease over a 24-months period. Median (M) and Interquartile range (IQR) are shown.

	AD converters	AD non-converters	p value
CD14+/TLR8+	17.9 (16.6–19.1)^a^	36 (22.7–38.3)	**0.0004**
CD14+/TLR9+	23.9 (19.6–25.9)^a^	43.3 (41.9–48.1)	**0.0001**

^a^TLR8 and TLR9 MFI calculated on MFI-positive cells alonen.

### Pro-and anti-inflammatory cytokine mRNA expression in Aβ_1-42_ stimulated PBMC

**Figure 2:**
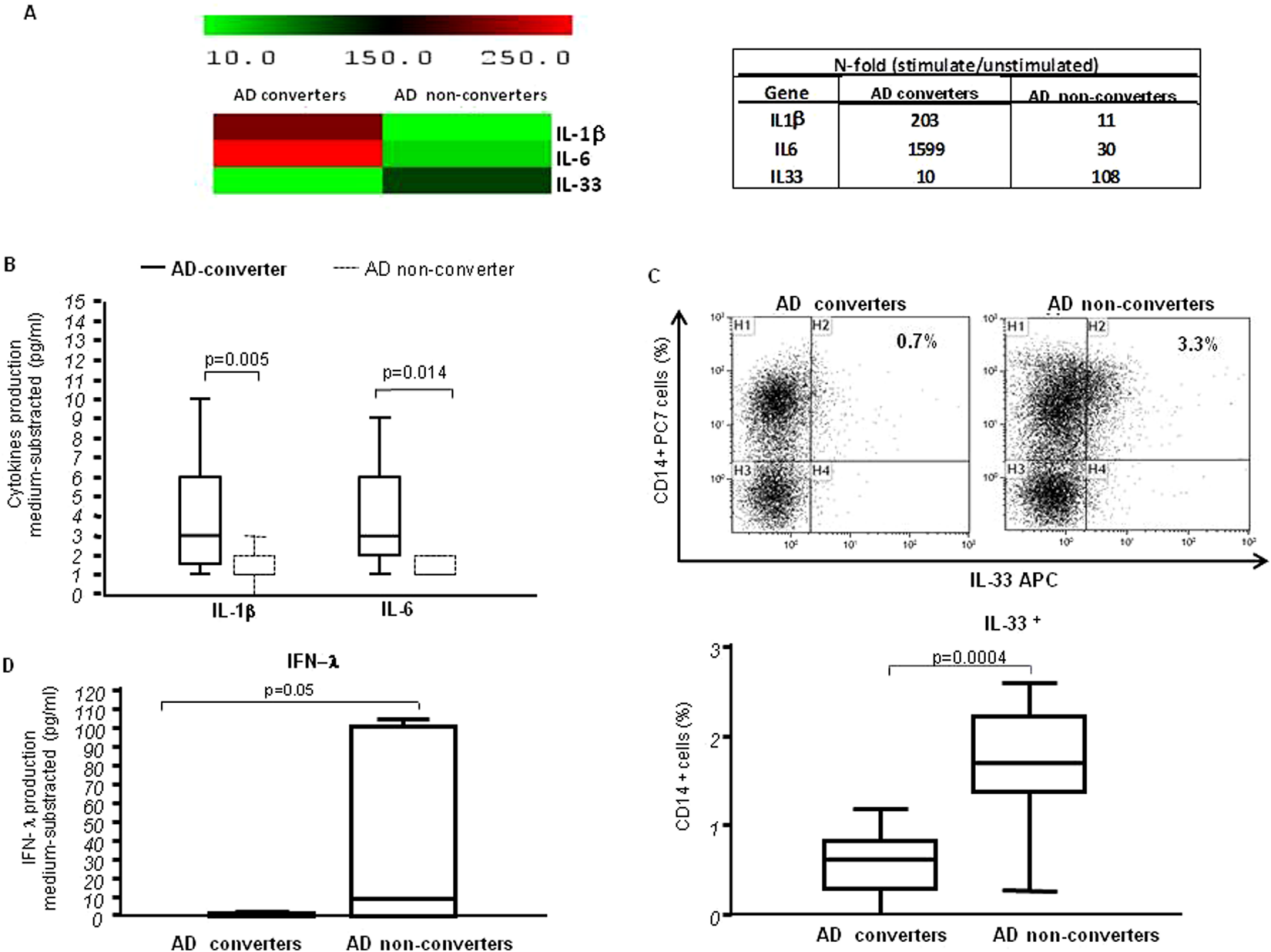
Pro-and- anti-inflammatory cytokine mRNA expression and protein in stimulated cells: (**A**) Aβ_1-42_-stimulated IL-1β, IL-6 and IL-33 mRNA expression in MCI individuals who did (AD converters, N = 21) or did not (AD non-converters, N = 25) convert to AD over a 24-months period. Gene expression was calculated relative to GAPDH housekeeping gene. The expression of the genes is assessed by single real-time quantitative RT-PCR and shown as median of fold-change expression from the unstimulated samples. Summary results are shown in the bar graphs using TIGR Multi Experiment Viewer (MeV)v4.9. Aβ_1-42_-stimulated IL-1β and IL-6 production (**B**); IL-33 expressing CD14^+^ cells (%) (**C**), and IFN-λ production (**D**) in PBMC of MCI individuals who did (AD converters; N = 21) or did not (AD non-converters; N = 25) convert to AD over a 24-months period. The boxes stretch from the 25 to the 75 percentile; the line across the boxes indicates the median values; the lines stretching from the boxes indicate extreme values. Outside values are displayed as separate points. Statistical significance is shown.

Quantitative PCR analyses were performed in PBMC that were either cultured in medium alone or were stimulated with Aβ_1-42_ (Fig. 2A). Results obtained in Aβ_1-42_– stimulated cells indicated that IL-1β and IL-6 mRNA was greatly reduced in AD non-converters compared to AD converters (IL1β > 200 fold; IL6 > 1000 fold; p = 0.04 and p = 0.05 respectively). In contrast, no significant results (n-Fold change < 2) were obtained for IFN-λ 1,2,3 mRNA expression. Results also showed that IL-33 mRNA was robustly (>100 fold) increased in AD non-converters (Fig. 2A).

### Pro-and anti-inflammatory cytokine production by Aβ_1-42_ -stimulated PBMC

Cytokine production was measured by ELISA in supernatants of PBMC that were either unstimulated or were stimulated with Aβ_1-42_. Once again, no differences were observed in unstimulated conditions (data not shown). When Aβ_1-42_- stimulated IL-1β and IL-6 production was measured, nevertheless, results showed that both these pro-inflammatory cytokines were significantly reduced in AD non-converters, compared to AD converters (p = 0.005 and p = 0.014 respectively) (Fig. 2B). These results support the idea that the presence of an inflammatory milieu associates with AD conversion in individuals with a diagnosis of aMCI.

**Figure 3:**
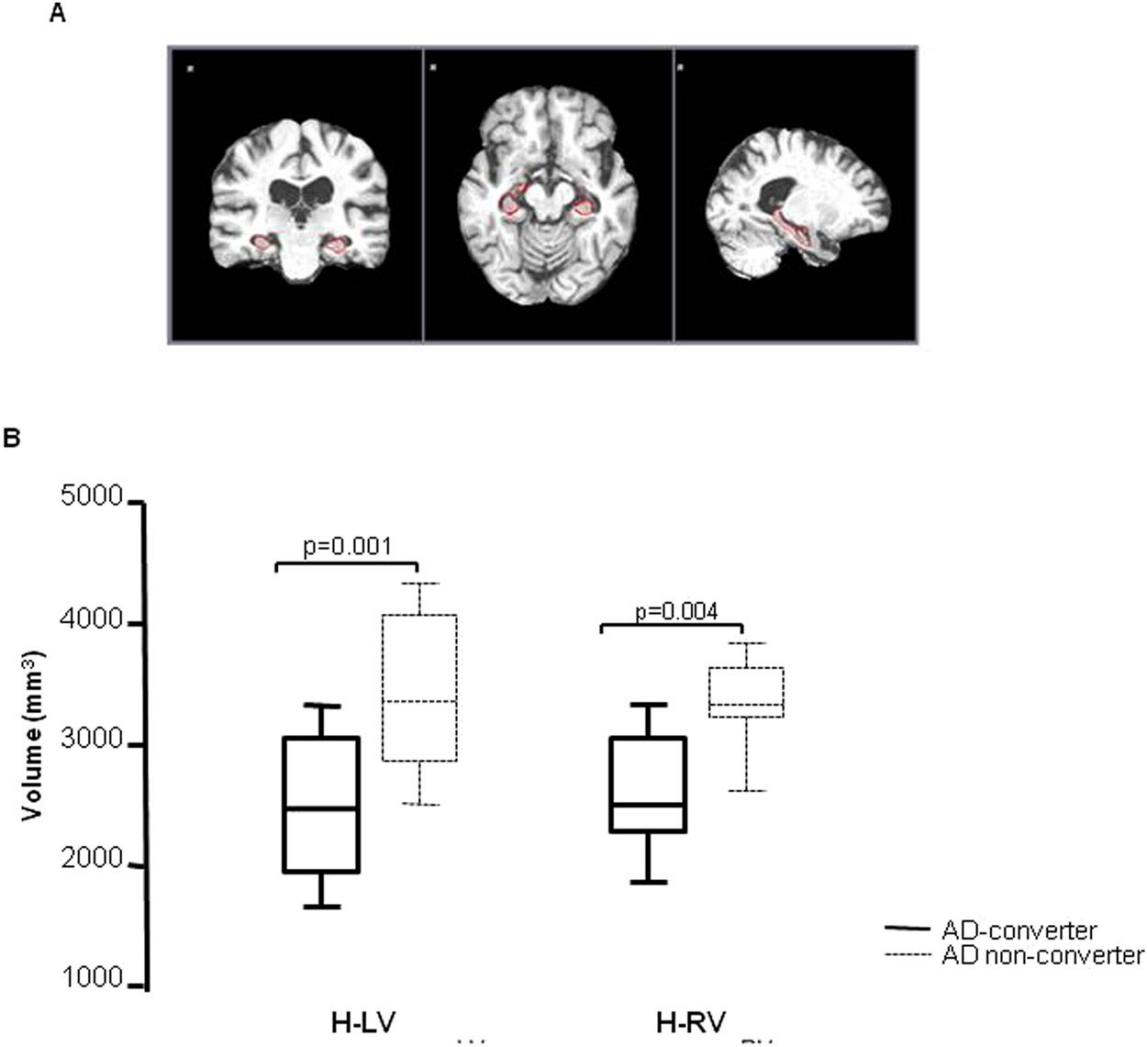
Brain mask of aMCI-typical Hippocampus-region and MRI analysis: an example of automatic hippocampal segmentation using the ADABoost algorithm. Coronal, axial and sagittal brain views in a single subject are shown in the upper part (**A**); left (LH) and right (RH) hyppocampal volumes as evaluated by MRI in MCI individuals who did (AD converters, N = 13) or did not (AD non-converters, N = 14) convert to AD over a 24-months period. The boxes stretch from the 25 to the 75 percentile; the line across the boxes indicates the median values; the lines stretching from the boxes indicate extreme values. Outside values are displayed as separate points. Statistical significance is shown (**B**).

IL-33 could not be measured in supernatants of Aβ_1-42_-stimulated PBMC, possibly because the mature form of this cytokine is not cleaved and secreted. Results obtained by flow-cytometry, nevertheless showed that CD14^+^/IL-33^+^ cells were significantly increased in AD non-converters compared to AD converters (p = 0.0004) (Fig. 3B).

### Antiviral cytokines: IFN-λ 1–3 production by Aβ_1-42_- stimulated PBMC

IFN-λ 1–3 production by unstimulated and Aβ_1-42_- stimulated PBMC was measured in all the individuals enrolled in the study. Results showed that Aβ_1-42_- stimulated IFN-λ 1–3-production was higher in AD non-converters compared to AD converters (p = 0.05), suggesting that the ability to mount robust antiviral immune responses correlates with lack of progression to AD (Fig. 3C).

### MRI results and correlations

High-resolution structural MRI acquisition was available at baseline in 27 aMCI individuals, 13 of whom did (5 males, mean age 75 ± 6.3 years) and 14 of whom did not (6 males, mean age 73 ± 7.1 years) progress to AD. The two “MRI subgroups” (AD converters and AD non-converters) were comparable for age (two sample t-test, p = 0.4785) and gender (chi-square, p = 0.8731) at baseline. MRI volumetric measurements at baseline showed a trend indicating a better preservation of hippocampi volumes in AD non-converters, with a stronger effect when the right hippocampus was analyzed (AD non-converters *vs*. AD converters, left hippocampus (H-LV), F(1,27) = 13.7, p = 0.001 (and right hippocampus (H-RV), F(1,27) = 17.25, p = 0.004 (Fig. 3B).

**Figure 4:**
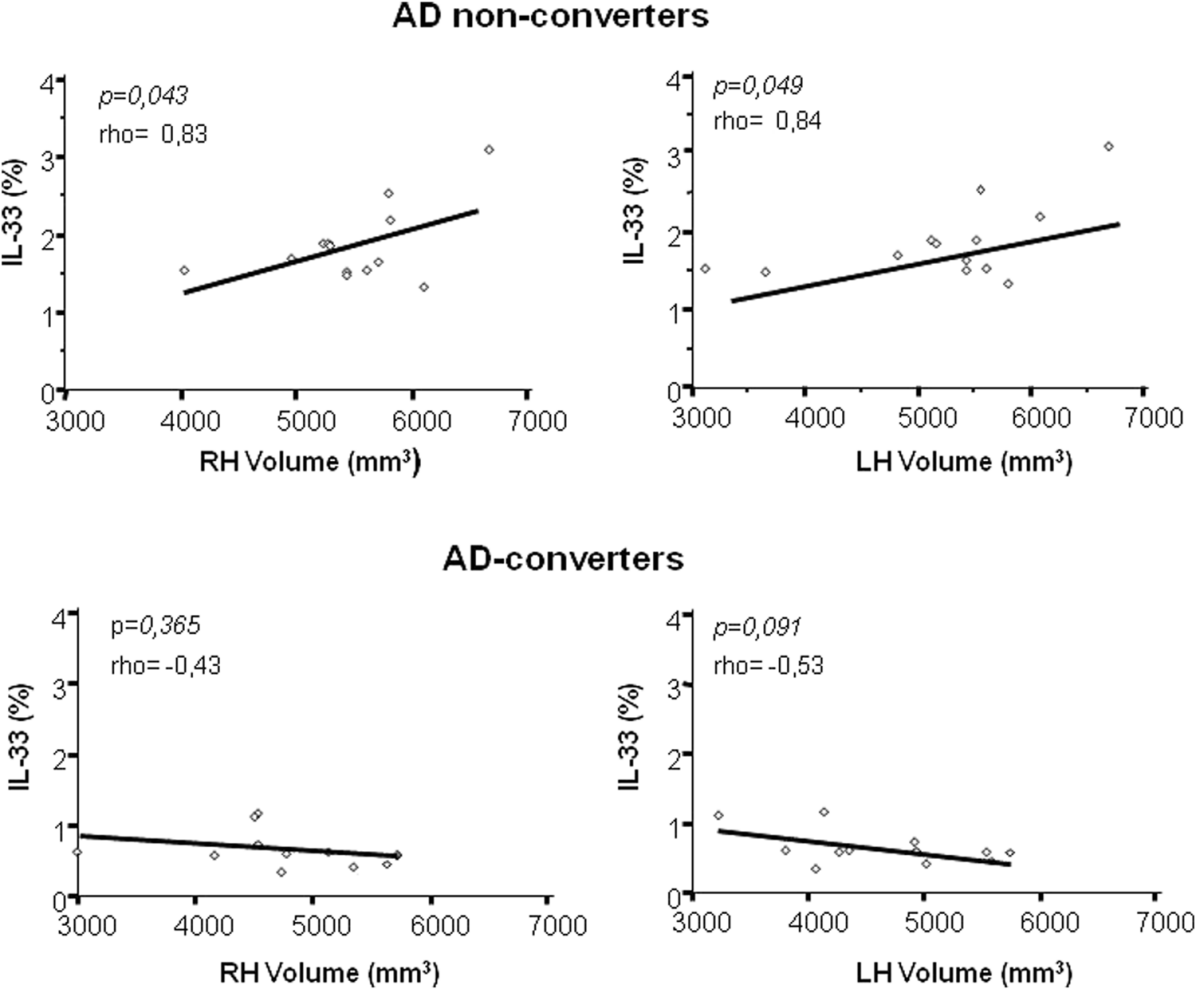
MRI analysis and correlations: Rank correlation between Aβ_1-42_ stimulated IL-33 producing CD14^+^ cells and the Left (L) and Right (R) hippocampus volume (HV) in MCI individuals who did (N = 13) or did not (N = 14) convert to AD. Statistical significance and Spearman’s coefficient of rank correlation (rho) are shown.

Notably, the analyses of correlations indicated a significant positive association between CD14^+^/IL-33^+^ cells and the volumes of both left and right (p = 0.049 and p = 0.043 respectively) hippocampus in AD non-converters alone (Fig. 4). No significant differences were found when correlations between MRI results and other immunological parameters were evaluated.

## Discussion

In mild cognitive impairment a slight but noticeable and measurable decline in cognitive abilities can be detected. Individuals with a diagnosis of MCI, and in particular those with a diagnosis of amnestic MCI (aMCI), have an increased risk of developing AD. Clinical, biological and imaging markers predicting MCI conversion to AD are still poorly clarified, possibly because the etiopathogenesis of AD itself is still barely understood. We analyzed immunologic and imaging markers in two groups of aMCI individuals who did or did not convert to AD over a 24 months period. We described herein a combination of these markers that might be useful in predicting which patients will convert to AD.

We focused on imaging analyses of MRI-based measures of hippocampal volumes, one of the core diagnostic criteria for probable AD dementia^2^; thus we verified whether this parameter could be used as a biomarker to assess the presence of downstream neuronal degeneration or injury. Notably, hippocampal volume was recently suggested to be a possibly adequate index for the screening of subjects at risk of developing AD^19,31^.

Results showed that, besides being associated with a peculiar cytokine profile, lack of MCI conversion to AD in our aMCI individuals was also correlated with better preserved hippocampal volumes, with a more pronounced effect in the right hippocampus. Importantly, better preserved hippocampal volumes were associated with higher percentages of circulating CD14+/IL-33^+^ cells in AD non-converters alone, suggesting a role for this cytokine in contrasting progression to AD in individuals with a diagnosis of aMCI. It is worth remarking that our analysis was adjusted for age, considering that increased variability in cognition with age has been argued as an indication of pathological processes^32^. These results support data indicating that MCI affects the size of the hippocampus, and that this parameter, together with the rate of hippocampus shrinkage, can be useful in predicting whether MCI does progress to AD^33^.

Viral infections, and in particular reactivation of HSV-1 infection^34,35^ are suggested to be involved in the pathogenesis of AD^34^; this hypothesis was recently reinforced by data indicating that accumulation of Aβ in the brain, the hallmark of AD, could play an antimicrobial role^36^. Immune defenses against viruses are initiated by ligation of TLR3, 8 and 9 by products of viral replication; this results in the production of cytokines with inflammatory and antiviral properties^37^. We and others have observed that the expression of TLRs that are engaged by viral PAMPs is up-regulated in CD14^+^ immune cells of MCI individuals^30,38^ possibly suggesting that stronger antiviral responses could be present in MCI. As a confirmation of this hypothesis, we show herein the presence of a significantly higher density of TLR8 and 9 on CD14+ cells in MCI patients that did not converter to AD, suggesting that a higher expression of viral PAMPs-specific immune sensors is associated with lack of disease progression.

Amongst the cytokines that were differentially modulated in AD converters and AD non-converters, and that are part of TLR-stimulated innate immune responses, IFN-λ stood out. IFN-λ includes at least three slightly different proteins that trigger mechanisms that contribute to the clearance of viral infections^39,40^ and, in particular, potently target HSV-1^41,42^. We recently described the presence of a positive correlation between HSV-1-specific serum antibody titers and grey matter volumes in the brain area that are classically affected by AD^43^. It is thus tempting to speculate that stronger IFN-λ-mediated antiviral responses could play a role in avoiding progression to AD by impeding the excessive reactivation of viruses that persistently infect the CNS, possibly including HSV-1.

AD has been associated with neuroinflammation as the production of a number of pro-inflammatory cytokines is increased in cells of AD patients^44^. We observed that Aβ_1-42_-stimulated production of IL-1β and IL-6, in particular, discriminate between those aMCI individuals who progress to AD, and those in whom such progression is not observed. IL-1β has long been known to play a role in the pathogenesis of AD^41,45^. This cytokine promotes amyloid plaque deposition, induces a loss of phagocytic activity by the microglia, stimulates the hyperphosphorylation of τ protein, and affects synaptic plasticity^46^. As a consequence, IL-1β can impair learning and memory processes. IL-1β also recruits peripheral leukocytes to the brain parenchyma^45^. Peripheral monocytes are able to directly cross the BBB, both generating novel microglial cells and potentially further extending chronic neuroinflammation^47^. Since these cells are considered to be better at amyloid plaque removal than resident microglia^48^, increases in IL-1β production were suggested to be a -possibly futile- attempt by the brain parenchyma to control β-amyloid accumulation. IL-6 concentration is also known to be increased both in animal models of AD and in patients^49,50^. Aβ directly stimulates the generation of IL-6 by human cortical neurons, playing a role in the production and the processing of amyloid precursor protein (APP). IL-6 also plays an important role in regulating cognitive function. Thus: i) elevated IL-6 correlates with age-related cognitive decline in humans, and ii) excessive IL-6 production alters spatial learning and memory^51,52^. Our results indicate that higher IL-1β and IL-6 production is detected in aMCI individuals converting into AD, reinforcing the idea that increased quantities of these inflammatory cytokines play a deleterious role in the development of AD.

IL-33 was also analyzed; this cytokine belongs to the IL-1β family and is a dual function protein with both intra and extra-cellular mechanisms of action. IL-33 can interact with the transcription factor NF-KB reducing NF-KB-triggered gene expression to dampen pro-inflammatory signalling^53^. Nuclear IL-33 can also induce the expression of intercellular and of vascular adhesion molecules for endothelial cell activation though binding to the p65 promoter^54^. Thus, IL-33 may have a role in regulating pathophysiology and inflammatory responses in the CNS^55^. IL-33 is reduced in AD brains, and this cytokine is believed to have a neuroprotective role secondary to the reduction of Aβ peptides secretion and the activation of their phagocytosis by the microglia^56^. The complexity of this cytokine is further underlined by recent results showing that three different polymorphism within the IL-33 gene resulting in a protective haplotype are associated with risk of AD^56^. Notably, recent results showed that in the APP/PS1 mouse model of AD IL-33 polarizizes microglia/macrophage toward an anti-inflammatory phenotype and reduces the expression of proinflammatory cytokines, including IL-1β and IL-6^52,57^. Indeed our results indicate that increased amounts of IL-33 correlate with lower production of IL-1β and IL-6 in AD non-converters. Taken together, these results suggest that higher amounts of IL-33 in aMCI individuals in whom AD conversion is not observed is a successful strategy to reduce neuroinflammation.

All aMCI patients enrolled in the study were selected after retrospective analysis of a cohort of MCI patients; selection was based on the availability of neurocognitive evaluation at baseline and after 24 months, and of peripheral blood cells (PBMC) samples and brain MRI at baseline. Although the overall validity of our data is somewhat limited by the small number of patients analyzed, these results suggest that neuroinflammation, possibly associated with weaker antiviral responses, plays a fundamental pathogenetic role in AD.

## Methods

### Patients

This study was approved by and carried out in accordance with the guidelines of the ethic committee of the Don Gnocchi Foundation. All participants or, if unable, their care-givers, gave informed consent to a protocol approved by the local ethics committee according to a protocol approved by the local ethics of the Don Gnocchi Foundation. The study conformed to the ethical principles of the Helsinki Declaration.

A large cohort of MCI individuals is followed by the Unit of Rehabilitative Neurology at the Don C. Gnocchi Foundation in Milano, Italy; a portion of these individuals is affected by amnestic MCI (aMCI). The diagnosis of aMCI fulfills Grundman’s and Petersen’s operational criteria^7,58^. Within the aMCI cohort two homogeneous groups of subjects (21 AD converters and 25 AD non-converters) were selected for the study. Inclusion criteria were based on: a) availability of a clinical follow up for at least 24 months; b) neurocognitive evaluation (MMSE) at baseline as well as after follow-up; c) availability of PBMC samples at baseline; and d) availability of a 3D single high-resolution structural MRI acquisition at baseline in addition to conventional MRI scans (in this case data were available in 13 AD converters and 14 AD non- converters) at baseline. The clinical diagnosis of AD was performed according to the NINCDS-ADRDA work group criteria^2^. The study conformed to the ethical principles of the Helsinki Declaration. All patients or, if unable, their care-givers, gave informed consent to a protocol approved by the local ethics committee.

### Blood sample collection and cell separation

Whole blood was collected by venipuncture in EDTA-containing Vacutainer tubes (Becton Dickinson & Co, Rutherford, NJ, USA). Peripheral blood mononuclear cells (PBMCs) were separated on lymphocyte separation medium (Ficoll-Hypaque, Organon Teknika Corp, Durham, NC, USA) and washed twice in PBS; viable leukocytes were determined using a TC20 Automated Cell Counter (Biorad Hercules,California, USA), PBMC were frozen and cold-preserved in liquid nitrogen in a cryoprotective media containing 10% dimethyl sulfoxide (DMSO) and fetal bovine serum (FBS). When needed for the experiments PBMC were thawed at 37 °C, washed in PBS and resuspended in RPMI 1640 to ensure optimal PBMC viability (>90%) for the cell culture.

### Cell cultures

PBMC (1 × 10^6^/ml) were cultured in RPMI 1640 supplemented with 10% human serum, 2 mM L- glutamine, and 1% penicillin (Invitrogen, Ltd, Paisley, UK) and incubated at 37 °C in a humidified 5% CO_2_ atmosphere for 24 h. PBMC were stimulated with 10 μg/ml synthetic oligonucleotides containing unmethylated CpG dinucleotides (ODN); 2 μg/ml single-stranded RNA oligonucleotide (ssRNA); or Aβ_1-42_ (10 μg/ml)(Sigma-Aldrich), St. Louis, MO or medium alone. Supernatants were used for cytokine detection; PBMC pellet were used for flow-cytometry analyses.

### ApoE genotyping

Genomic DNA was isolated from whole blood by phenol-chloroform extraction. Customer-design Taqman probes for the 112 and 158 codons were used to determine the genotype of apolipoprotein E gene (APOE)^43^.

### RNA extraction and reverse transcription

RNA was extracted from unstimulated or Aβ_1-42_ stimulated-PBMC and reverse transcribed into first-strand cDNA^59,60^. Real Time quantitative Reverse Transcription PCR (RQPCR) was performed using the ABI Prism 7000 instrument (PE Applied Biosystems, Foster City, CA, USA) with gene specific primers and SybrGreen chemistry described elsewhere^59,60^. The following cytokines were analyzed: IL-1β, IL-6, IL-33 (Qiagen, Hilden Germany) and IFNλ 1, 2, 3 (Sino Biological, LuDong Area, BDA China). Specific PCR products amplification was detected using the RT2 SYBR Green Fluor with a 25 μl final volume of 12.5 μl RT_2_ qPCR Mastermix (Qiagen), 10.5 μl H_2_O, 1.0 μl of either diluted template RT_2_ qPCR Primer Assay. Results were expressed as ΔΔCt and presented as ratios between the target gene and the Glyceraldehyde 3-phosphate dehydrogenase (GAPDH) housekeeping mRNA. Experiments were individually run on each one of the individuals included in the study.

### Flow cytometry immunofluorescent staining

PBMC were stained with anti-CD14-PC7 (clone RMO52, isotype mouse IgG_2a_, Beckman Coulter) and treated with the FIX and PERM Cell kit (eBioscience, San Diego, CA) before being stained with TLR8-PE (clone 610015, isotype mouse IgG_1_, R&D Systems), TLR9-FITC (clone M9.D6, isotype rat IgG_2a_, eBioscience), or IL33-APC (clone 390412, isotype rat IgG_2b_, R&D Systems) specific mAbs.

### Flow cytometry

Analyses were performed using a Beckman-Coulter Cytomics FC-500 flow cytometer equipped with a single 15 mW argon ion laser operating at 488 nm and interfaced with CXP Software 2.1 and Beckman-Coulter GALLIOS flow cytometer equipped with a 22 mW Blue Solid State Diode laser operating at 488 nm and with a 25 mW Red Solid State Diode laser operating at 638 nm, and interfaced with Kaluza analysis software. Green florescence from FITC was collected through a 525-nm bandpass filter, orange-red fluorescence from PE was collected through a 575-nm bandpass filter and blue fluorescence from PC7 was collected through a 770-nm bandpass filter excited by 488 nm laser (FC-500); far-red fluorescence from APC was collected through a 660/20-nm bandpass filter excited by 638 nm laser (GALLIOS). Two-hundred-thousand cells were acquired and gated on lymphocyte and monocyte forward and side scatter properties. Data were collected using linear amplifiers for forward and side scatter and logarithmic amplifiers for fluorescences. Samples were first run using isotype control or single fluorochrome-stained preparations for color compensation. Rainbow Calibration Particles (Spherotec, Inc. Lake Forest, IL) were used to standardize flow-cytometry results in samples obtained over time.

### Measurement of cytokines: ELISA

IL-1β, IL-6, IL-33 and human Interferon-lambda (IFN-λ)1–3 concentration was measured in supernatants of either unstimulated or Aβ_1-42_-stimulated PBMC by multiplex sandwich immunoassays according to the manufacturer’s recommendations (Quantikine Immunoassay; R&D Systems). Briefly, 200 μl per well of standard, sample, or control were transferred into the IL-1β, IL-6 or IFN-λ antigens coated polystyrene microwells and the plates were incubated for 2 hours at room temperature. After three washing steps with washing buffer to remove the unbound proteins, 200 μl of Human IL-1β IL-6 or IFN-λ peroxidase conjugate was added to each well and incubated at room temperature for 2 hours. After re-washing step, 200 μl of chromogen/substrate solution were added to each well and incubated at room temperature for 20 min protect from light. Finally, 50 μl of stop solution were added to each well and the reaction stopped. The wells were read on a plate reader (Sunrise, Tecan, Mannedorf, Switzerland) and optical densities (OD) of wells were determined at 450/620 nm. The measured absorbance is proportional to the concentration of cytokines (IL-1β or IL-6) and IFN-λ present in the supernatants expressed in pg/ml and calculated by dividing optical density (OD) measurement generated from the assay by OD cut-off calibrator. All the experiments were performed in duplicate. Sensitivity (S) and Assay Range (AR) were as flows: S: IL-1β = 1 pg/ml, IL-6 = 0,7 pg/ml and IFN-λ = 50ng/ml; AR: IL-1β 3.9–250 pg/ml, IL-6 3.1–300 pg/ml, IFN-λ = 62,5–4000 ng/ml.

### MRI analysis

Hippocampal volume data have been extracted for each subject from high-resolution T1 3D images collected using a 1.5T scanner (Siemens, AVANTO) at the time of MCI diagnosis - MPRAGE; TR/TE = 1900/3.37 ms, FoV = 192 mm × 256 mm, in-plane resolution 1 mm × 1 mm, slice thickness = 1 mm, number of axial slices = 176) using AdaBoost (see Fig. 3A), a fully-automated machine-learning segmentation algorithm^61,62^. ADABoost is a fully-automated machine-learning segmentation algorithm implementing the EADCADNI harmonized hippocampal segmentation protocol for Alzheimer-related pathologies, able to automatically segment subcortical structures as hippocampus. Hippocampal volumetries were compared across the two groups (AD-converters and AD non-converters) at baseline and were also used to compute regression analyses with anti-inflammatory cytokines production.

### Statistical analysis

Quantitative data were not normally distributed (Shapiro-Wilk test) and are thus summarized as median and interquartile range (IQR; 25° and 75° percentile). Firstly, comparisons between two groups (AD converters and non converters) were analyzed to evaluate immunological differences; comparisons were made using a 2-tailed Mann-Whitney U test for independent samples. Data analysis was performed using the MedCalc statistical package (MedCalc Software bvba, Mariakerke, Belgium).

Secondly, we performed Kruskal-Wallis ANOVA models, adjusted for MMSE to compare right and left hippocampal volume levels in the two groups of AD converters and AD non-converters. This analysis was performed using the package “sm” of the R software, freely available. Thirdly, separately in each group of AD-converters and AD non-converters, we calculated the correlations (using Spearman’s coefficient) between hippocampal volume levels and other immune parameters. P-values < 0.05 are reported as statistically significant in the text. Power was calculated with the G-power software^63^. Notwithstanding the small samples, effect sizes were reasonably large and attained power was above 0.90 for all comparisons.
